# Supplementary material for: Utility of Clinical Next Generation Sequencing Tests in KIT/PDGFRA/SDH Wild-Type Gastrointestinal Stromal Tumors
Source: Cancers (Basel). 2024 Apr 27;16(9):1707. doi: 10.3390/cancers16091707 (PMC11083047; doi:10.3390/cancers16091707)
Supplement: Supplementary file 1 [file cancers-16-01707-s001.zip › cancers-2942643-supplementary.pdf]

cancers

MDPI

Table S1. Review of all the cases of triple or quadruple negative GIST reported in the literature.

Ref	Age at Diagnosis	Sex	Primary Site	Primary Tumor Size (cm)	Mitotic rate (per 50 hpf or 5 mm2)	Disease State at Diagnosis	Presumed Driver Mutation
Current study	41	Male	Gastric	22	50	IIIB	*PTEN* deletion
Current study	68	Female	Small intestine	4.5	69	IV	?
Current study	38	Male	Small intestine	2.5	0	IA	*FGFR1* GOF
Current study	21	Female	Gastric	7	0	IB	?
Current study	17	Female	Gastric	6.5	3	IV	?
Current study	12	Female	Gastric	NA	20	?	*BRAF* V600E
Current study	57	Female	Small intestine	5.7	12	IB	?
Current study	44	Male	Gastric	12	75	IV	*NTRK3* fusion
Current study	37	Male	Gastric	30	8	IV	?
Current study	72	Male	Small intestine	5	18	IV	*NF1* deletion
Current study	58	Male	Small intestine	15	44	IIIB	*BRAF* V600E
Current study	48	Female	Small intestine	6	15	IIIA	?
Current study	14	Female	Gastric	NA	NA	NA	*CHEK2* LOF
Current study	73	Male	Small intestine	8.9	156	IV	Aurora A fusion
Current study	50	Female	Gastric	7	NA	NA	*NF2* splice site mutation, *FGF3/FGF19* amplification, *CCND1* amplification, *TP53* LOF
Current study	58	Female	Gastric	2	50	II	*NF1* splice site
Current study	54	Female	Colorectum	5.2	8	IIIA	*FANCA* deletion
Current study	53	Male	Small intestine	9.5	2	IV	*TP53* LOF
Current study	25	Female	Gastric	14.5	10	IV	?
Current study	11	Female	Gastric	NA	NA	NA	?
[19]	NA	NA	NA	NA	103	NA	*BRAF* V600E
[19]	NA	NA	NA	NA	0	NA	*BRAF* V600E
[19]	NA	NA	NA	NA	NA	NA	*BRAF* V600E
[19]	NA	NA	NA	NA	18	NA	*NF1* indel/frameshift (pH2240fs)
[19]	NA	NA	NA	NA	147	NA	*NF1* indel/frameshift (p.17_17del, p.G629R)
[19]	NA	NA	NA	NA	2	NA	*NF1* missense (p.E775G)
[19]	NA	NA	NA	NA	160	NA	*CBL* missense (p.P417H)
[19]	NA	NA	NA	NA	42	NA	*KIT-PDGFRA* fusion
[19]	NA	NA	NA	NA	20	NA	*ARID1A* missense (p.M918I)
[19]	NA	NA	NA	NA	<5	NA	?
[19]	NA	NA	NA	NA	2	NA	?
[20]	NA	NA	NA	NA	NA	NA	*NF1* nonsense (p.Q959X)

[20]	NA	NA	NA	NA	NA	NA	*NF1* nonsense (p.Q519X)
[20]	NA	NA	NA	NA	NA	NA	*NF1* nonsense (p.R1241fs)
[34]	57	Male	Small intestine	NA	NA	Localized	?
[34]	63	Female	Small intestine	NA	NA	Localized	?
[42]	44	Male	Colorectum	5	34	NA	*ETV6-NTRK3* fusion
[42]	86	Female	Small intestine	8	8	NA	?
[42]	26	Female	Small intestine	5	10	NA	?
[42]	71	Female	Small intestine	10	8	NA	?
[42]	31	Female	Small intestine	9	20	NA	?
[18]	58	Female	Small intestine	6	2	NA	?
[18]	35	Male	NA	NA	NA	NA	*NF1* nonsense
[18]	76	Female	Small intestine	1.5 + 2.5	1	NA	*NF1* splice acceptor variant
[18]	69	Female	Small intestine	NA	21	NA	*TP53* LOF
[18]	73	Female	Small intestine	8	18	NA	*NF1* deletion
[18]	53	Male	Retroperitoneum	8	20	NA	?
[18]	60	Female	Small intestine	6	2	NA	*NF1* nonsense and frameshift mutations
[18]	31	Female	Small intestine	9	83	NA	*NF1* nonsense
[18]	73	Female	Peritoneum	16	30	NA	*NF1* missense variant
[18]	77	Female	Gastric	15	29	NA	?
[18]	59	Male	Small intestine	1.5	0	NA	*NF1* frameshift and splice donor mutations
[18]	50	Male	Small intestine	6	1	NA	*NF1* splice acceptor and donor mutations
[18]	86	Female	Small intestine	8	8	NA	*NF1* nonsense
[18]	68	Female	Gastric	5.5	5	NA	?
[18]	68	Male	Small intestine	2.3	1	NA	?
[18]	70	Female	Small intestine	11.5	87	NA	?
[18]	41	Female	Colorectum	8	39	NA	*NF1* missense variant
[18]	56	Male	Gastric	3.2	8	NA	*NF1* frameshift variant
[18]	67	Female	Small intestine	3	2	NA	*NF1* deletion and frameshift mutations
[18]	60	Male	Small intestine	4.5	3	NA	?
[18]	36	Male	Gastric	11	10	NA	*NF1* frameshift mutation
[18]	30	Female	Small intestine	3.2	3	NA	?
[22]	52	Female	Small intestine	10	90	Primary	*BRAF*
[22]	55	Female	Small intestine	10	5	Primary	*BRAF*
[22]	59	Female	Small intestine	9	50	Primary	*BRAF*
[22]	66	Male	Peritoneum	NA	NA	Relapsed	*BRAF*
[49]	70	Male	Gastric	0.4	<5	Primary	*BRAF*
[49]	80	Male	Small intestine	0.4	<5	Primary	*BRAF*
[50]	53	Male	Small intestine	20	6	Primary	*BRAF*
[50]	38	Male	Small intestine	2.5	5	Primary	*BRAF*

[50]	63	Male	Gastric	2.5	NA	Primary	*BRAF*
[50]	78	Male	Gastric	NA	1	Primary	*BRAF*
[50]	51	Female	Small intestine	3	10	Primary	*BRAF*
[50]	58	Male	Small intestine	2.5	1	Primary	*BRAF*
[50]	58	Male	Small intestine	2.5	6	Primary	*BRAF*
[50]	41	Male	Small intestine	2.5	3	Primary	*BRAF*
[50]	50	Female	Peritoneum	2.8	50	Primary	*BRAF*
[31]	NA	NA	Small intestine	NA	NA	Primary	*BRAF*
[31]	NA	NA	NA	NA	NA	Primary	*BRAF*
[32]	60	Male	NA	15	6	Primary	*BRAF*
[33]	75	Male	Peritoneum	NA	8	Relapsed	BRAF
[24]	69	Male	Small intestine	4.6	4	Primary	*BRAF*
[24]	36	Female	Small intestine	8.5	3	Primary	*BRAF*
[24]	66	Female	Small intestine	5.4	8	Primary	*BRAF*
[24]	63	Male	Small intestine	11.2	12	Primary	*BRAF*
[24]	42	Female	Small intestine	3.8	7	Primary	*BRAF*
[24]	89	Female	Small intestine	1.8	1	Primary	*BRAF*
[36,39]	63	Female	Small intestine	5-10	6-10	Distant	*CTNND2* LOF, *FGF4* amplification
[36]	73	Female	Small intestine	12	100	Localized	*TP53*
[36,39]	50	Male	Small intestine	8.5	2	Localized	*NF1, MAX*
[36]	41	Female	Colon	8	80	Distant	*NF1*
[36,39]	73	Male	Small intestine	13	<5	Localized	*MEN1, TP53, FGF4* amplification
[36,39]	45	Female	Small intestine	NA	NA	Localized	*CDH4, FGF4* amplification
[36,39]	45	Male	Small intestine	NA	NA	Localized	*FGFR1*
[39]	57	Male	Small intestine	1.6	<5	Localized	*FGF4* amplification
[39]	69	Male	Small intestine	NA	NA	Localized	*FGF4* amplification
[39]	44	Male	Small intestine	6.5	>5	Distant	*FGF4* amplification
[23]	71	Female	Esophagus	2.8	< 5	NA	*BRAF*
[23]	68	Female	Gastric	10.5	2	NA	*BRAF*
[23]	60	Female	Small intestine	8.0	< 5	NA	*BRAF*
[25]	54	Male	Small intestine	NA	5-10	NA	*NF1* frameshift
[37]	38	Female	Small intestine			T3N1M1	*FGFR1-HOOK3 fusion*
[37]	54	Male	Gastric			T3N1M1	*FGFR1-TACC1 fusion*
[37]	55	Male	Small intestine			T3N0M1	*ETV6-NTRK3* fusion
[37]	60	Male	Small intestine			T3NxM1	*FGFR1* missense mutation
[37]	72	Male	Small intestine			T4N1Mx	*FGF6* amplification
[37]	54	Male	Pelvic mass				*FGFR1-TACC1 fusion*
[37]	54	Male	Colorectum				*ETV6-NTRK3* fusion
[37]	49	Male	Small intestine				?
[37]	51	Female	NA				?
[37]	53	Male	Gastric				?

NA, not available; ? indicates that no potential driver alteration was identified.; For the statistics from this table that are described in the text, ranges were recoded as the average of the upper and lower bounds, < 5 was recoded as 0, and > 5 was recoded as 5.
